# The Concept of Creating Digital Twins of Bridges Using Load Tests

**DOI:** 10.3390/s23177349

**Published:** 2023-08-23

**Authors:** Marcin Jasiński, Piotr Łaziński, Dawid Piotrowski

**Affiliations:** Faculty of Civil Engineering, Silesian University of Technology, 44-100 Gliwice, Poland; marcin.jasinski@polsl.pl (M.J.); piotr.lazinski@polsl.pl (P.Ł.)

**Keywords:** digital twin, building information modeling, bridge, FEM models, load tests

## Abstract

The paper sheds light on the process of creating and validating the digital twin of bridges, emphasizing the crucial role of load testing, BIM models, and FEM models. At first, the paper presents a comprehensive definition of the digital twin concept, outlining its core principles and features. Then, the framework for implementing the digital twin concept in bridge facilities is discussed, highlighting its potential applications and benefits. One of the crucial components highlighted is the role of load testing in the validation and updating of the FEM model for further use in the digital twin framework. Load testing is emphasized as a key step in ensuring the accuracy and reliability of the digital twin, as it allows the validation and refinement of its models. To illustrate the practical application and issues during tuning and validating the FEM model, the paper provides an example of a real bridge. It shows how a BIM model is utilized to generate a computational FEM model. The results of the load tests carried out on the bridge are discussed, demonstrating the importance of the data obtained from these tests in calibrating the FEM model, which forms a critical part of the digital twin framework.

## 1. Introduction

Modern industrial development is inseparably linked to the digitization and automation of processes [1,2]. Information technology systems that replace or aid human activities contribute to improving reliability and productivity in the industry. At the same time, they reduce the probability of mistakes, especially those arising from repeatability, time-consumption, requirement to maintain constant attention or scope, diversity, and complexity of data appearing at various stages of the decision-making process. One of the most advanced IT systems designed to solve these difficulties is the concept of a digital twin [3,4]. Research and work on its implementation are carried out in many fields, including robotics and manufacturing [5,6], energy [7], mining [8,9], agriculture [10], logistics, specifically road [11], rail [12], watercraft [13], and aircraft transportation [14,15], or in medicine [16]. The intensive development of this technology also applies to the construction industry, including civil and bridge engineering [17,18,19].

In all these fields, the definition of digital twin is derived from common assumptions, based on a virtual copy of an existing object, which allows its ongoing monitoring [20]. The creation of the twin is usually preceded by the creation of a digital model [21]. The link between the model and the real object can be one-way, referred to as a digital shadow, or two-way, forming the framework of the digital twin. The one-way link may consist of collecting and storing data in the model, without ongoing evaluation, with an independent and limited impact on the decision-making processes related to the object. Extending it to the two-way form requires, in addition to collection, additional analysis of the data, its visualization, and automated processing by inference, control, and warning systems [22]. In the construction industry, it can be achieved using structural health monitoring [23,24] aided by novel sensing and digitization techniques such as laser scanning or photogrammetry [25,26], Internet of Things (IoT) [27], machine learning, and artificial intelligence [28], as well as virtual reality (VR), mixed reality (MR), or augmented reality (AR) [29]. Digital twins based on these elements were successfully developed and tested as components of tools for asset management systems [30,31], damage detection [32,33,34,35], and performance optimization [36].

**Figure 1:**
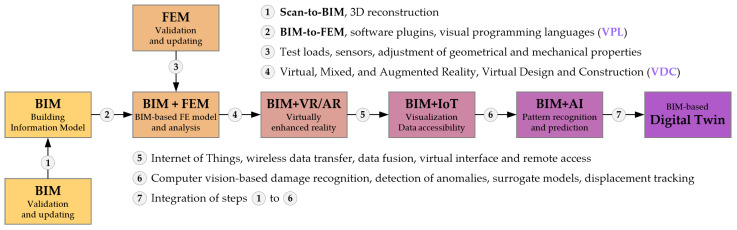
Transition of the BIM model to the digital twin, using auxiliary techniques and validation processes.

Usually, a digital twin of a building is an enhanced version of a model following the Building Information Modeling (BIM) paradigm [37]. The BIM model, as a basis for the digital twin, can be supplemented with auxiliary techniques and tools to form a faithful reproduction of the building, sensitive to its ongoing changes (Figure 1). The BIM model can be created and developed at the design stage, e.g., to generate drawings, quantity take-offs, or schedules. At the same time, it also provides the information required in the subsequent stages of the construction process. In the operational phase, by linking it to the sensor system, it serves as an interface of the digital twin, allowing visualization of the measured values and providing access to archival data. Modifications to the existing structure resulting from the twin analysis are also introduced back into the BIM model. In the design and operational stages, the usefulness of the BIM model can be extended to generate a finite element analysis (FEM) model which, in addition to the BIM counterpart, plays an equally crucial role in the whole system. The essence of the digital twin is to measure the loads and responses of the structure and then compare the results with the computational FEM-based simulation. The comparison is performed automatically and affects decisions and strategies in the management process. However, it is not possible to perform a reliable analysis without updating the BIM and FEM models to ensure their compliance with the real structure.

Validation and updating of the BIM model can be achieved using laser scanning and other noncontact measurement techniques, namely, Scan-to-BIM, to capture the as-built geometry and physical state of the structure [38]. Compliance between real and computational responses requires validation tests performed under controlled conditions with comparison between measured and simulated output. The load test is one of the most widely used methods to evaluate the performance of existing structures, especially bridges [39]. Among the most common testing patterns, static and dynamic identification tests are generally performed, based on displacement and vibration measurements, respectively [40]. Currently, load testing is the most accurate method for assessing bridge capacity [41]. It can be used as part of an acceptance procedure for newly constructed bridges [42,43,44] and old structures [45,46], as well as a direct method of performance evaluation in strengthened or repaired bridges [47,48,49]. Obtaining high compliance of the FEM model in terms of span stiffness and dynamic response requires updating the initial model where the characteristics were assumed or imposed by design standards. Static tests allow for verification of the flexural stiffness of the spans, the load distribution between the elements of the superstructure, and the settlement of the supports. In the case of concrete structures, the stiffness depends on various factors, including the type of aggregate, the age of the concrete, and the curing process, and can be affected by the influence of the nonstructural elements. Dynamic tests are used to determine the real response related to the modes and the corresponding vibration frequencies, damping, and acceleration of the span. They depend on its stiffness, the weight of the structure, nonstructural elements, and vehicles on the bridge, as well as the environmental conditions. The data obtained from the experimental evidence are used to generate an updated FEM model that should be used in the process of creating digital twins.

The idea of generating the FEM model based on the BIM model or point cloud data was investigated in numerous studies, especially for heritage sites [50,51,52]. It includes software-specific transformation rules, solid-based finite element models, plugins, or scripts based on visual programming interfaces. The latter is particularly useful in various engineering design tasks known for their specificity, uniqueness, and complex forms. Visual programming, namely, Dynamo or Grasshopper, was successfully used to generate bridge FEM models in Midas Civil and Matlab engineering software [53]. Jia et al. [54] described an automatic generation of FEM models based on BIM and ontology, analyzing an open IFC (Industry Foundation Classes) for spatial data. Textural programming languages, including Python, was used to generate FEM models of tunnels [55] and dams [56] basing on their BIM models. Similar methods can be used in the digital twin framework, yet the model itself should still be validated and updated to ensure enough compliance with the real structure. Thanks to this, once the construction stage is completed, the management system is enriched with the as-built BIM model associated with the updated FEM model. Further data collected in the structure life cycle can be compared with the output of the verified FEM model to automatically decide or alert about the current state. Obtaining and processing information can minimize the risks associated with the use of critical infrastructure and reduce the maintenance costs incurred by the administration units.

The purpose of this paper is to define the role of a load testing procedure in the process of creating a digital twin. Load tests are well-known and common experimental evidence performed in bridge engineering long before the concept of digital twins was even established in the construction industry. The significance of the field tests and their results is highlighted and presented in the example of a one-span concrete bridge. The issue is particularly relevant, as the load tests and proper updating of the FEM model for the purpose of creating digital twins seem to be omitted or marginalized in the literature on the subject. The paper presents the use of the bridge BIM model to generate a computational FEM model and its updating based on the results of the load and dynamic tests. The key issues apparent in the update process are discussed, showing possible inaccuracies that should be solved and adjusted to obtain the valid FEM model to be used as part of the digital twin.

## 2. Materials and Methods

Creating a digital twin of a building, also a bridge, is a continuous and multithreaded process. It includes the analysis of the structure conducted in a digital environment, before and after construction, as well as repeated verification tests on the real structure. Its final purpose is to measure the object’s response to loads and impacts, perform immediate computational simulation, and conduct comparative analysis between the measured and simulated output.

**Figure 2:**
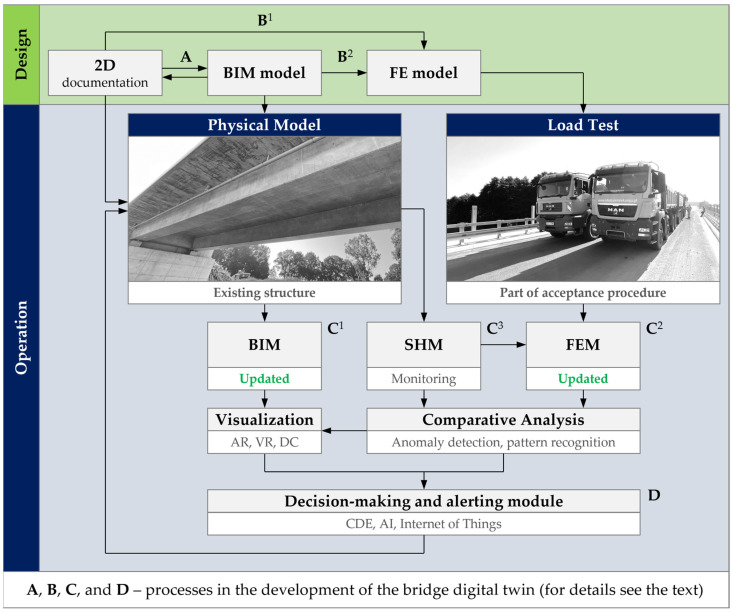
Digital twin framework with BIM model, BIM-based FEM model, and load test.

In the described approach, the BIM model is the basis of the digital twin. Considering its final purpose in the operational stage, including the use of augmented reality and other spatial visualization techniques, an important feature of the model is its three-dimensionality and accuracy, both on geometric and semantic levels. The BIM methodology enforces the appropriate enrichment of the model with data, especially the classification of members and elements by their physical and structural role in the building. They are complemented by metadata, geometric and material characteristics of individual components, relationships between them, and links to external databases. An example of such a database, particularly important in the concept of the digital twin, is the FEM model. In the case of existing structures, where the design process was carried out without implementing the BIM methodology, it may be necessary to create the BIM model from scratch, based on archival documentation, usually 2D, or physical inventory. For the latter, the laser scanning, photogrammetry, and other imaging techniques can be used. All the issues related to creating the BIM model based on the documentation, as well as generating the drawings from the model, are presented in Figure 2 by a two-way relationship A: 2D documentation ↔ BIM model.

An important digital twin component is the FEM model. Its creation can be carried out independently of the BIM model (Figure 2, relation B^1^) or with its direct use (Figure 2, relation B^2^). Due to the fact that both models, BIM and FEM, are parts of the final twin-based system, the desirable approach is to link the members of the computational model with the components of the BIM model. It may consist of the analysis of the topology of the model and the transformation of structural solid components into the corresponding 1D and 2D elements. The analysis and transformation in question can be carried out using tools that are part of BIM modeling software, as well as plugins written in textural or visual programming languages. The final products of the transformation are usually grillages (1D elements in 3D space), shells (2D elements in 3D space), or mixed models (1D and 2D elements combined in 3D space). In the case of bridges, an additional problem is limited compatibility between the BIM and FEM models, which prevents the use of generators built into the interface of the BIM modeling software. These models usually differ in the number of components, the relations between them, and the geometry in global and local terms. In the case of FEM models, the longitudinal alignment of the bridge is consciously neglected, so the vertical and horizontal curvature is reduced to series of straight-line segments. A single superstructure solid is then divided into longitudinal and transverse beam elements that do not appear explicitly in the BIM models. The role of transverse beams is generally to capture the impact of the slab on the load distribution between the girders. In the case of concrete–steel composite bridges, the tendency is the opposite and mainly consists of a reduction in the number of FEM components compared to the BIM model. The reduction is the result of combining the separate steel girder and the concrete slab into a single, all-steel transformed sections. For this reason, it is recommended to build FEM models based on the individual set of rules that supplement the interface of BIM software as plugins defined with the use of programming languages.

The mentioned relations describing the creation and analysis of the BIM model, the FEM model, and the documentation are usually limited to the design stage and allow for building a real structure, referred to here as the physical model. The concept of a digital twin is to associate the digital models with the physical model in such a way that all the entities influence each other. A necessary step towards this goal is verification and updating of digital models that can be coupled to the monitoring system of the existing structure. Updating the BIM model consists of adjusting the geometry of individual components, the relations between them, and their attributes. The descriptive layer is supplemented here with important events recorded during the construction stage. The final BIM model can serve as the as-built model, and its creation and verification can include reconciliation with a physically acquired inventory point cloud (C^1^ in Figure 2).

Verification and updating of the FEM model require experimental evidence on the real structure, especially including load and dynamic tests (C^2^ in Figure 2). Span deflections, settlements, and dynamic characteristics that are measured in situ, by comparison with the values simulated in the FEM model, allow for the adjustment of the basic features of the model to reconcile the response with the physical model. This is performed with a known and precisely applied load, often large enough to reveal possible non-linear characteristics of the structure’s response. Finally, the updated FEM model will be part of the digital twin, in which the ongoing structural analysis will be compared with the measurement of the Structural Health Monitoring system (SHM, C^3^ in Figure 2). Without adjustment, this comparison would remain incomplete, include disturbed results, and lead to invalid conclusions. The FEM model created in the design stage is characterized by various uncertainties that are impossible to capture or predict a priori. These include, for example, geometrical deviations, damping and modulus of elasticity of concrete, the influence of reinforcement, prestressing tendons and nonstructural elements on the stiffness of the span, conditions related to the foundation of the object, non-linearities, imperfections, or material inhomogeneity.

The updated FEM model is one of the components of the final digital twin. Measurements carried out on the existing structure are compared on an ongoing basis with the results of the FEM simulation. Due to the scope, amount, and complexity of the data, this comparison is usually carried out using automatic or semi-automatic systems based on machine learning and artificial intelligence algorithms [57,58,59]. It may include damage recognition based on the computer vision [60,61], structural condition assessment by pattern recognition and detection of anomalies [62], surrogate model-based reliability analysis [63], and dynamic measurement of displacement [64]. The results and decisions derived from the analysis are visualized in the as-built BIM model. It can be supplemented by merging the view of the existing structure with its digital representation, e.g., using virtual and augmented reality techniques. The whole output is stored and processed in a coherent, web-oriented environment, namely, CDE (Common Data Environment, D in Figure 2).

## 3. Generating FEM Models for the Digital Twins

Bridge structures are basic elements of infrastructure. To ensure the safety and durability of these structures, it is necessary to analyze their behavior under various load conditions. Increasing the efficiency and accuracy of this analysis is ensured by integrating the BIM methodology and FEM.

In the bridge design process, the BIM methodology provides an integrated environment for combining all information into one unified model: geometry, material properties, and structural components. This model becomes the basic source of data that can be used in the subsequent phases of the design and operation of the facility. The ability of BIM tools to store all information in one model makes them a promising basis for generating FEM models.

With the help of FEM, the complex geometry of the bridge is divided and discretized into smaller and easier to manage elements such as beams, slabs, or solid elements, where each of them is assigned specific properties, loads, and boundary conditions. To build an FEM model based on a BIM model, one should ensure they:

- Correctly export the bridge geometry while paying attention to topological differences between the BIM and FEM models;
- Discretize geometry into smaller finite elements;
- Assign appropriate material and cross-sectionals properties;
- Apply appropriate load schemes and boundary conditions.

**Figure 3:**
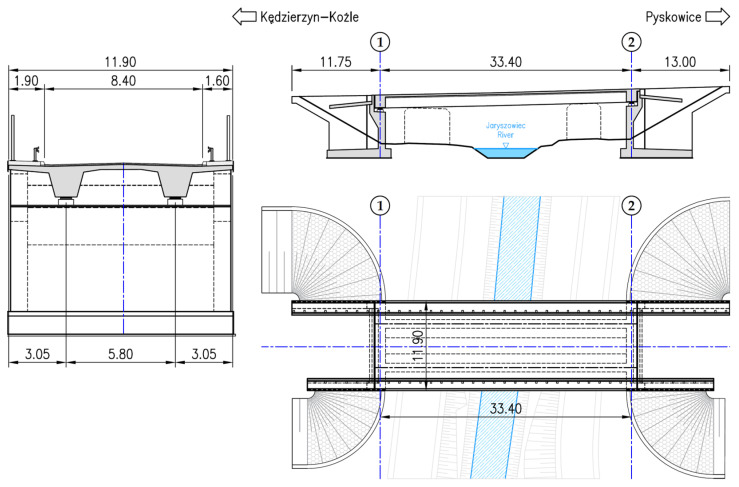
MD-18 bridge. General sections and the top view.

To present the issues of BIM-based FEM model creation, using it the process of validation and updating to comply with the results of a load test, the MD-18 bridge was used as an example (Figure 3). High compliance is required for the models to become an integral part of a digital twin. The bridge is an existing structure on the northern bypass of Kędzierzyn-Koźle, Poland, located along the national road no. 40. The object was completed in 2022.

The bridge is a single-span, simply supported beam made of C35/45 class prestressed concrete. The span length is 33.40 m and the total width is 11.90 m. A two-way roadway with a width of 8.40 m is led along the bridge, with raised footways, safety barriers, and screens on both sides. The superstructure consists of two beams with a trapezoidal cross-section with an axial spacing of 5.80 m, connected by a reinforced concrete slab with thickness of min. 0.24 m. The width of the beams at the bottom is 1.15 m, and their height is 1.65 m. The cantilevers on both sides have an overhang of 2.13 m and a variable thickness of 0.35 m when attached to 0.24 m at the end. Crossbeams with a width of 1.40 m and a height of 1.55 m have been designed in the support cross-sections. The spans are supported on two pot bearings on each support. The abutments were designed as massive and made of reinforced concrete. The end supports are located at an angle of 90.0° to the axis of the object. The structure was designed for road load class A + 0.3K according to the Polish standard [65].

**Figure 4:**
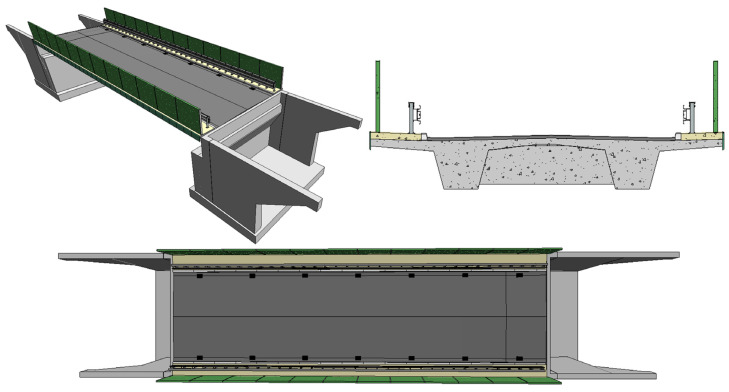
MD-18 bridge. Overview of the BIM model.

Models incorporating the finite element method and analysis (FEM) are an important element used in the process of designing bridges. On their basis, it is possible to analyze the behavior of the structure under various load conditions. The traditional approach to the design of bridge structures requires frequent changes to the FEM model through subsequent iterations, updating its geometry and loads. A wide range of data and parameters must be entered manually, making the process time-consuming and thus increasing the risk of mistakes occurrence. Hence, it is reasonable to implement an alternative approach to automate the generation of FEM models. The BIM model of the bridge presented in Figure 4 was adopted as a starting point. On its basis, an analytical model was generated and used directly in the FEM environment. Such an approach significantly reduces working time and costs, as well as speed up decision-making in emergency situations, minimizing the risk of errors.

The geometry of each bridge in the BIM and FEM model is varied. While BIM models contain detailed information about the geometry, FEM models require their simplified version to perform the analysis effectively. Hence, the transformation between these two models cannot be performed directly and requires some modifications. For the described object, the target FEM model consisting of 1D and 2D elements combined in a 3D space should be prepared, in which the girders and the deck slab are represented by 1D elements, which form the grillage. In the BIM model, the superstructure was created using a single longitudinal beam without cross members. To generate the FEM model, this geometry was analyzed, modified, and supplemented using scripts made in the Dynamo visual programming language (VPL), enabling the integration of the structural data between the BIM and FEM environments.

**Figure 5:**
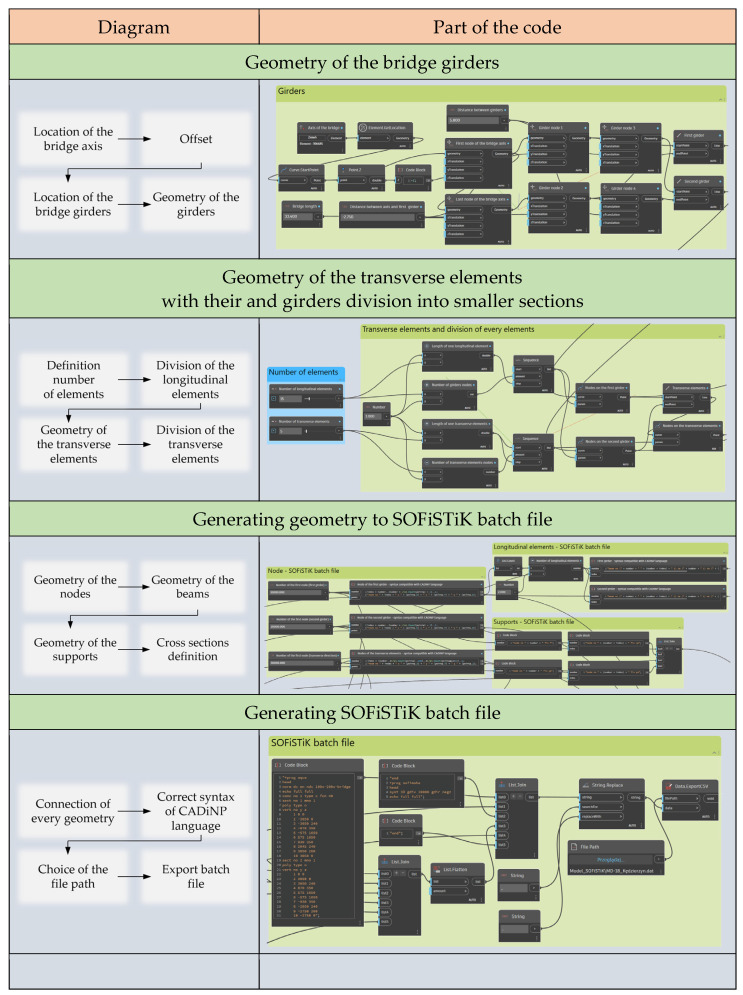
Generating the FEM model for SOFiSTiK software using Dynamo visual programming.

For this purpose, using Dynamo, a single beam of the superstructure modeled in the BIM environment was copied to a distance equal to half the girder spacing on each side. With this operation, the longitudinal bars of the girders were generated. The next step was to divide the created members into smaller sections, adding the grid division parameter in the longitudinal direction. This procedure allowed, first, the digitization of the girder beams and, second, the introduction of nodes enabling the addition of transverse elements. Similar to the girders, the transverse bars were also discretized, with their number depending on the division parameter in the transverse direction. The final part was to generate the geometry of all elements and supports for the SOFiSTiK batch file. The procedure is shown in Figure 5.

**Figure 6:**
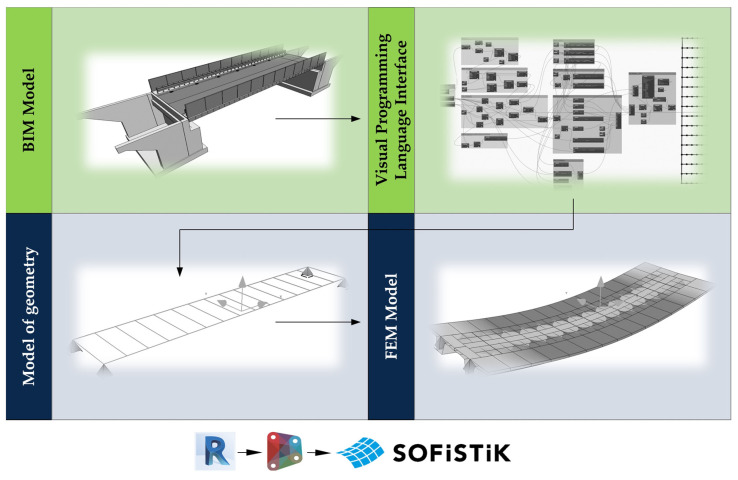
Exchange of information between the BIM and FEM models using the visual programming environment.

In the analyzed example, the FEM model was made in the SOFiSTiK software. Using the Dynamo script, a batch file was generated to preprocess Teddy of the MES software, based on the CADiNP language syntax (Figure 6). This approach has many advantages. One of the most significant is the ability to parameterize the model. In the script developed, two editable input parameters were introduced: the division of longitudinal and transverse elements into sections. The use of the Dynamo visual interface, when changing any parameter, allows for automatic generation of a new batch file and importing the geometry directly to the FEM model. This model can then be supplemented and modified, including creating the required load cases.

The generated FEM model consists of a geometry-, material-, and load model.

The geometry model based on the as-built documentation takes into account all geometric parameters with an accuracy of 1 cm—in this case, there is no significant simplification that would deteriorate the accuracy of the obtained results.

In the material model, standard features before the update were used, but then the model was updated using parameters based on in situ tests. This approach (shown in the next section) eliminates sources of uncertainty related to the material used.

In the load model, loading vehicles were weighed and their actual position on the object during load tests was taken into account. In this way, the uncertainty associated with the applied load is eliminated.

In the case of support, boundary conditions related to rotations and displacements of bearings were assumed in the neutral axis of the cross-section. The bearings used are characterized by low friction resistance and do not significantly affect the deterioration of the accuracy of the model.

## 4. Load Test on the Bridge and Its Results

**Figure 7:**
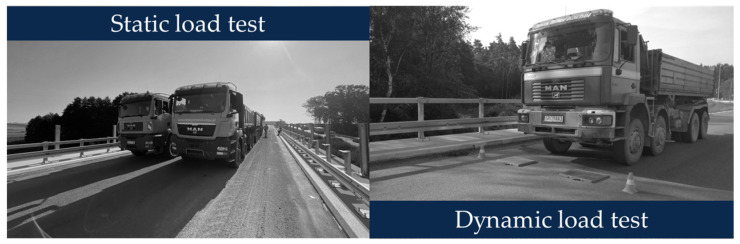
Static and dynamic load test of the MD-18 bridge.

The load tests performed on 17 August 2022 included static and dynamic tests. Static tests were carried out with the use of six trucks loaded to a total weight of 32 t each. In the dynamic tests, one vehicle was used, moving in both directions along specific paths and across prepared obstacles (Figure 7).

In the span, there was one load scheme implemented, selected from the condition of the maximum span moment. At the same time, displacements were measured in both the span structure and each of the supports. This was carried out until the displacements stabilized, according to the accredited testing procedure of the quality management system used by the Research Team of the Silesian University of Technology, Poland. The procedure introduces assumptions of the ISO/IEC 17025 and ISO 9000 standards in the measurement of settlement, deflection, displacement, acceleration, and strain of bridge structures. It establishes the necessary definitions, operations, and notations for the proper registration of the bridge response during static and dynamic load tests. In case of span displacement, the procedure assumes the initial measurement after introducing the test load scheme on the bridge. The next series, not less than three, are carried out every 10 to 15 min until the deflection is stabilized. Stabilization is assumed if the deflection read in subsequent measurements does not differ more than 2%. If such a state is achieved, the test load is removed and the remaining deflection is measured until stabilized.

**Figure 8:**
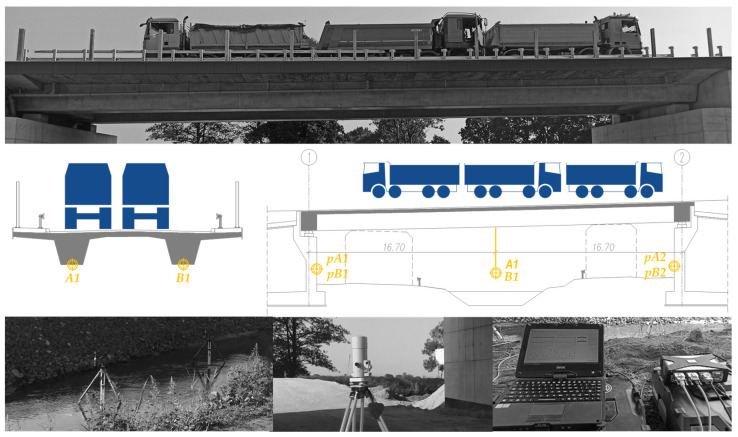
Implemented static load diagram with the arrangement of measurement points.

During static tests, mechanical sensors and precise levelling were used to measure displacements in a static load test. Vertical displacements of the span at points A1 and B1 in Figure 8 were measured with mechanical sensors with a range of up to 50 mm and a reading resolution of 0.01 mm. The settlement of the supports at the points pA1, pB1, pA2, and pB2 was observed using an optical precision leveler with a reading resolution of 0.1 mm.

Dynamic tests were performed using inductive sensors. Results in the form of time series of displacements were recorded electronically using the Siemens LMS Scadas Mobile (Gliwice, Poland) Recorder and inductive linear displacement sensors with a resolution of 0.01 mm and a sampling frequency of 300 Hz. The arrangement of the measurement points was in accordance with Figure 8. The recorded time series were processed using a 20 Hz low-pass digital Bessel filter.

**Figure 9:**
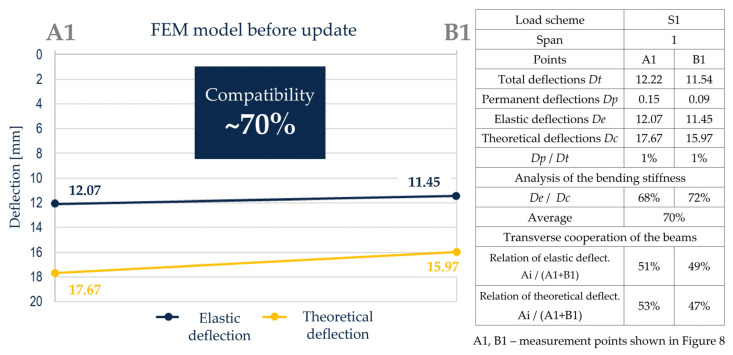
Analysis of the bending stiffness of the span and transverse cooperation of the beams based on measurements and in an unverified FEM model.

The elastic deflection values obtained during the measurements, listed in Figure 9, were compared with the computational deflections obtained in the unverified FEM model. Elastic deflections of the span were lower than those calculated theoretically and ranged from 68% to 72% of their value. On average, elastic deflections of the tested span accounted for about 70% of the calculated values. Such results demonstrate the greater stiffness of the span and the feasibility of the FEM model adopted for the design, giving safe results. Differences in the bending stiffness of the span are caused, among others, by the use of aggregate characterized by lower deformability relative to the standard assumptions. Additionally, a significant impact of bridge nonstructural elements, reinforcement, and prestressing steel was found, which is usually not taken into account in the design stage.

The implemented span load scheme was asymmetric, so it can be used to assess the transverse load distribution between the girders. The combined results in the form of temporary deflections under two beams were compared with the deflections calculated in the FEM model. The transverse distribution, defined here as the percentage of deflections of individual beams to their total deflections (Figure 9), shows that the distribution is slightly greater than that obtained in the model. The obtained results indicate the correct distribution and its high compliance with the calculation results.

**Figure 10:**
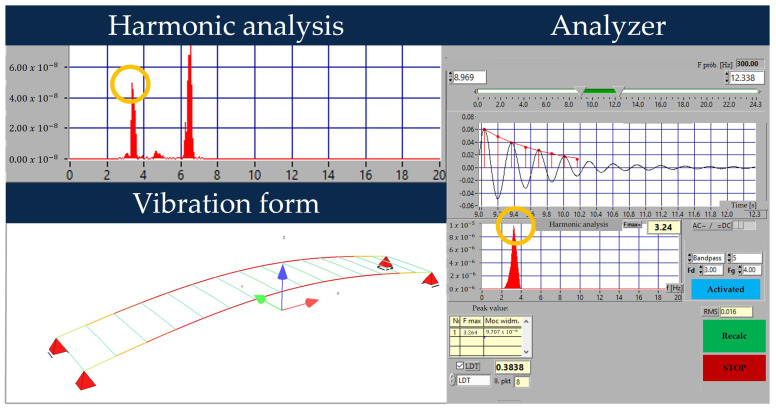
Identification of natural frequencies and corresponding damping.

Dynamic tests allowed the identification of two frequencies of free vibrations and compare them to the calculated values obtained in the unverified FEM model. The lowest frequency of the normal mode (bending vertical) was 3.24 Hz and was higher than the theoretically calculated 2.34 Hz (Figure 10). The average logarithmic decrement value was 0.3838, a typical moderately high value. The frequency differences obtained result from the increase in flexural stiffness confirmed in static tests.

The discrepancies found in the bending stiffness require the process of updating the FEM model. The following three phenomena that affect the increase in the stiffness of the span were taken into account:

- The impact of the aggregate and environmental conditions on the change in the modulus of elasticity of concrete determined on the basis of laboratory tests carried out using dedicated procedure [66];
- The impact of nonstructural elements determined on the basis of comparison with the technical documentation and own research assuming 72% of the footway area;
- The impact of reinforcement and prestressing determined by the change in the moment of inertia, based on the reinforcement ratio specified in the technical documentation.

The design stress–strain (σ–ε) relationship depends on many factors, including the composition of the concrete, curing conditions, and its age. The fact that the deformability of concrete varies depending on the type of aggregate is taken into account when determining the modulus of elasticity. It can be determined on the basis of laboratory tests or by introducing a value depending on the type of aggregate into the compressive strength formulas. In the case of the analyzed object, basalt aggregate from the Gracze mine was used, which is classified as a low-deformation aggregate.

**Figure 11:**
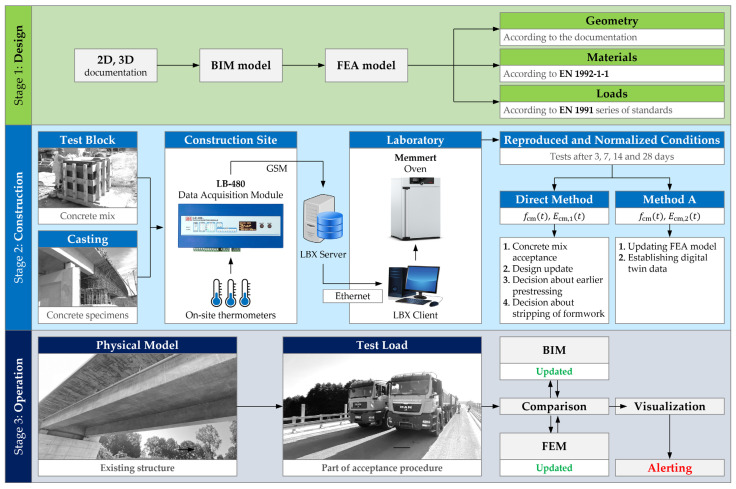
Identification of concrete deformability in the process of digital twin design and adjustment.

Identification of concrete deformability in the process of erecting concrete bridges requires the use of a method that also takes into account other factors affecting the final value of the modulus. The concrete recipe should include the specification of components that affect the early development of the mechanical properties of concrete, including the strength and modulus of elasticity, as well as the hydration heat. Taking into account the observations of contractors and designers, a method for identifying the deformability of concrete for decision-making purposes was proposed (Figure 11).

The research consists of two stages. The first stage should be carried out on a test block in order to make a decision on approval of the recipe, taking into account the construction technology and the related expectations of the designer and contractor. The dimension of the block should be determined individually based on the geometry of the main parts of the superstructure. The authors propose a block with dimensions of 1.20 m × 1.20 m × 1.20 m. The second stage includes the supervision of pouring. Obtaining conditions reflecting the concrete curing and its hardening is carried out using an innovative remote monitoring system. The system consists of temperature sensors placed in the test block and structure, the LB-480 recorder, the LBX server and the Memmert oven. By recording the temperature in the test block, the system controls the oven, obtaining similar conditions for the care of samples in the laboratory. The procedure allows obtaining the concrete of similar parameters as used and cured in the real structure. Its characteristics, especially the modulus of elasticity, can then be directly introduced to the FEM model in the digital twin.

**Figure 12:**
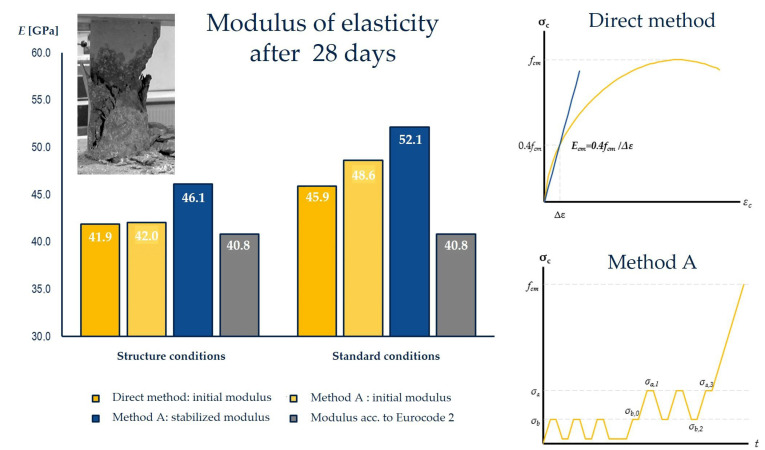
Summary of the modulus of elasticity of concrete determined by the direct method and method A after 28 days of curing in standardized conditions.

**Figure 13:**
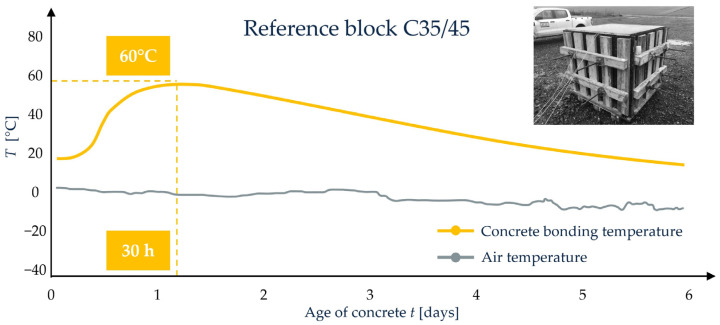
Measurement of the temperature of the hydration heat of the C35/45 concrete used in the test block for the curing of samples under simulated conditions.

The analysis of the increase in concrete strength and the modulus of elasticity is presented in Figure 12. The modulus of elasticity of the concrete class C35/45 assumed in the design was 37.8 GPa according to the Polish standards [65], which corresponds to the value of 34.0 × 1.2 = 40.8 GPa according to Eurocode standards [67,68]. For the purposes of updating the FEM model in the operational stage, after the elimination of permanent deformations, the values of the modulus of elasticity of concrete determined by Method A, according to standard [69], were tested on samples after 28 days of curing under simulated conditions (Figure 13). The value of the modulus obtained is 46.1 GPa. The resulting 15% increase in the stiffness of the structure can be taken into account by entering the value in the model of the material.

The standardized Method A was used to determine the secant modulus of elasticity of concrete with the elimination of permanent deformations. It corresponds to the use situation in which the bridge spans are subjected to cyclical impact.

Prestressed structures have already eliminated permanent deformations, where the cross-section is subjected to a change in compressive stresses in the linear range. This method determines the initial and stabilized modulus according to the loading process shown in Figure 12.

The direct method is related to one cycle of stress increase to failure. Determination of the secant modulus of elasticity of concrete is carried out without eliminating permanent deformations, which corresponds to the first load of hardened concrete during the process of stressing or removing scaffoldings.

The concrete test samples were cured in standardized conditions (stored in water) and in simulated conditions (stored in a dryer controlled by the concrete setting temperature). Both curing methods give different modulus of elasticity values [52].

**Figure 14:**
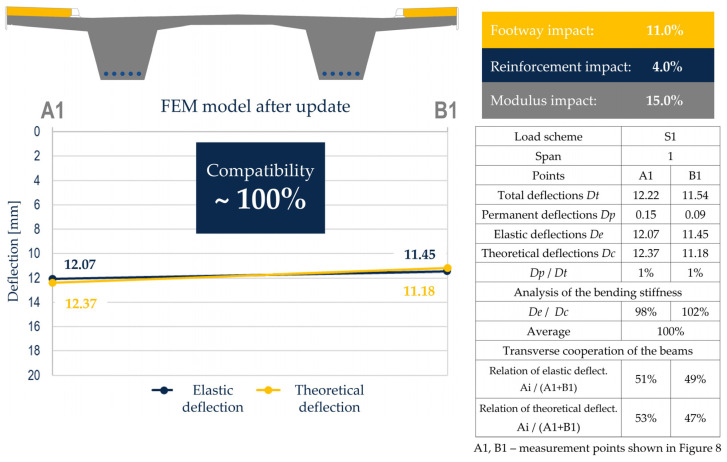
Analysis of the flexural stiffness of the span and the transverse cooperation of the beams based on measurements and in the verified FEM model.

Taking into account the reinforcement and impact of concrete footways, the increase in stiffness of 4% and 11% was introduced, respectively, by increasing the moment of inertia of the girders in the FEM model. Using the described update procedure, the verified model can be obtained with stiffness and eigenfrequencies consistent with the results on the tested span (Figure 14). This model can be used directly in the operational stage as an integral part of the digital twin for decision-making purposes.

During the update of the geometry model, the area of the reinforcement with the steel modulus of elasticity was introduced, which resulted in the increase in the modulus of inertia for bending. The 4% increment was quantified by calculation.

The 11% impact of the interaction of the footway with the structure was taken into account on the basis of own experimental research on objects where the structure was subjected to a load without and after making the footway.

## 5. Discussion

This article provides a comprehensive exploration of the digital twin concept as applied to bridge structures, highlighting its potential in the field of facility management throughout the life cycle of the bridge.

One of the primary contributions of this research lies in the exploration of load testing as a crucial step in the creation and validation of the digital twin for bridge structures. By subjecting the bridge span to static and dynamic impacts, the study successfully determined the response of the structure. This information is invaluable as it allows for a more accurate representation of the bridge behavior within the digital twin framework. Consequently, the results obtained from load testing enabled the identification of bending and torsional stiffness, as well as dynamic features, serving as essential parameters for updating the FEM Model.

Furthermore, the study recognizes the influence of nonstructural elements and concrete deformability on the performance of the bridge. By conducting thorough research on these aspects, it is able to incorporate high compliance into the digital twin framework. This inclusion further enhances the accuracy and reliability of the computational model, enabling a more realistic simulation of the behavior of the bridge.

The verified calculation model, established through the integration of load testing data and taking into account nonstructural elements and concrete deformability, has great potential for facility management throughout the entire life cycle of the bridge. The digital twin, which incorporates this validated model, offers numerous benefits in terms of monitoring and optimizing bridge performance. With real-time data integration, asset managers can make informed decisions about maintenance, repairs, and overall asset management strategies. This approach can improve safety and increase longevity, ultimately leading to more efficient and cost-effective management practices.

As the field of digital twins continues to advance, further research and practical applications will undoubtedly contribute to the ongoing development and refinement of this innovative approach to bridge asset management.

## Data Availability

On request from the authors.
